# Fitz-Hugh-Curtis Syndrome in a Male Patient

**DOI:** 10.7759/cureus.60749

**Published:** 2024-05-21

**Authors:** Ahmed Mostafa, Mohamad Alhalabieh, Justin Scarano, Sakshi Dhar, Omer Nasir

**Affiliations:** 1 General Surgery, Nazareth Hospital - Trinity Health Mid-Atlantic, Philadelphia, USA

**Keywords:** fitz-hugh-curtis syndrome, general surgery, sexually transmitted disease (std), perihepatitis, case report

## Abstract

We report the case of a 38-year-old Middle Eastern man with intractable right upper quadrant (RUQ) abdominal pain and several emergency department visits during the last seven years, with extensive and repeated radiologic and endoscopic workups proven negative for biliary or upper gastrointestinal disease. He presented to our outpatient surgical clinic in March 2023 complaining of worsening RUQ and epigastric pain and was scheduled for a robotic cholecystectomy for presumed biliary dyskinesia following a repeat cholescintigraphy (hepatobiliary iminodiacetic acid) scan. During a cholecystectomy, extensive bilobar perihepatic adhesions were found, indicative of Fitz-Hugh-Curtis syndrome (FHCS). A thorough lysis of adhesions was performed along with a wedge liver biopsy, with subsequent histological examination showing chronic cholecystitis, perihepatic mesothelial fibrosis with mild subcapsular hepatic steatosis, and no evidence of liver fibrosis. The patient was examined in the clinic two weeks after surgery with complete resolution of symptoms. This case highlights the importance of considering FHCS in the differential diagnosis of male patients presenting with refractory RUQ abdominal pain despite a negative workup. Early recognition and prompt treatment can prevent unnecessary extensive, repeat testing and delays in intervention in these patients.

## Introduction

Fitz-Hugh-Curtis syndrome (FHCS), also known as perihepatitis, is characterized by inflammation of the liver capsule and is thought to be a sequelae of pelvic inflammatory disease (PID). The liver capsule forms fibrin “violin string” adhesions to the surrounding abdominal wall and diaphragm, but the liver parenchyma is not affected. Presenting symptoms include right upper quadrant (RUQ) pain, fever, chills, vomiting, and malaise. FHCS is primarily found in women and is a result of sexually transmitted diseases (STDs) such as *Neisseria gonorrhoeae* and *Chlamydia trachomatis*. The incidence ranges from 4% to 14% in women with PID but is found to be higher in adolescents (27%), potentially due to less mature genitourinary tract. The pathogenesis by which these bacteria cause perihepatitis is not completely understood. Proposed mechanisms include an ascending infection from the cervix/vagina to the endometrium, fallopian tubes, and subsequently into the peritoneal cavity [1]. This could explain the disproportionately higher number of cases in women. Other potential causes include lymphatic spread into the parametrium from intrauterine devices and hematogenous spread. The diagnosis of FHCS is one of exclusion, ruling out other causes of RUQ pain. In this case report, we discuss a rare finding of FHCS in a male patient undergoing a robotic cholecystectomy.

## Case presentation

**Figure 1:**
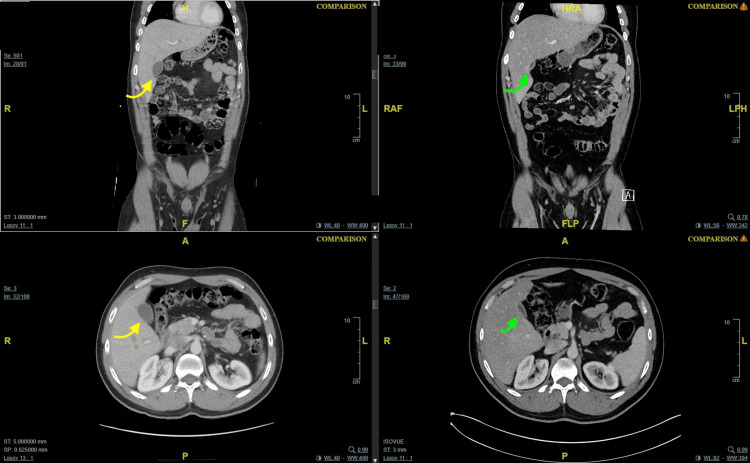
CT scan of abdomen and pelvis with a repeat in three years. The left upper and bottom panels represent the prior CT scans showing a distended non-inflamed gallbladder (yellow arrows). The right upper and bottom panels represent the latter CT scan showing a constricted non-inflammed gallbladder (green arrows). Both show no gallstones, wall thickening, or pericholecystic fluid suggesting acute cholecystitis.

A 33-year-old Middle Eastern man presented to the emergency department in a community hospital in Philadelphia in February 2018, with a diffuse pruritic scalariform rash. Laboratory workup was unremarkable, and he was discharged with a course of diphenhydramine and steroids. A month later, he presented with RUQ and epigastric tenderness and laboratory workup showed no significant findings. The abdominal ultrasound showed mild hepatic steatosis, with no gallbladder sludge or stones. A CT scan of the abdomen and pelvis with intravenous contrast was only significant for a 20 cm craniocaudal hepatomegaly. Gastroenterology was consulted, and after a negative viral hepatitis panel, he was sent home with an outpatient esophagogastroduodenoscopy, which showed normal healthy mucosa of the esophagus and stomach, with a negative *Helicobacter pylori* test on gastric biopsy. In April 2021, he presented to the emergency department with an eight-month history of burning upper abdominal pain and constipation. Repeat abdominal ultrasound and CT scan of the abdomen and pelvis with intravenous contrast were significant for hepatic steatosis and hepatomegaly with gallbladder contraction (Figure 1). The laboratory workup was again unremarkable, and he was discharged with an outpatient surgical referral.

The patient was examined in the surgical clinic in March 2023 with chronic episodic RUQ and epigastric pain, not related to meals or relieved with famotidine antacids. He had no previous abdominal surgeries. He underwent a hepatobiliary iminodiacetic acid scan with cholecystokinin showing an ejection fraction of 76% (normal gallbladder ejection fraction is >35%; regardless of age and gender) but complained of severe RUQ the same night of the procedure. He started reporting postprandial pain, which worsened with fasting. He was seen again at the clinic, and given he met the Rome III criteria, he consented to a robotic cholecystectomy.

**Figure 2:**
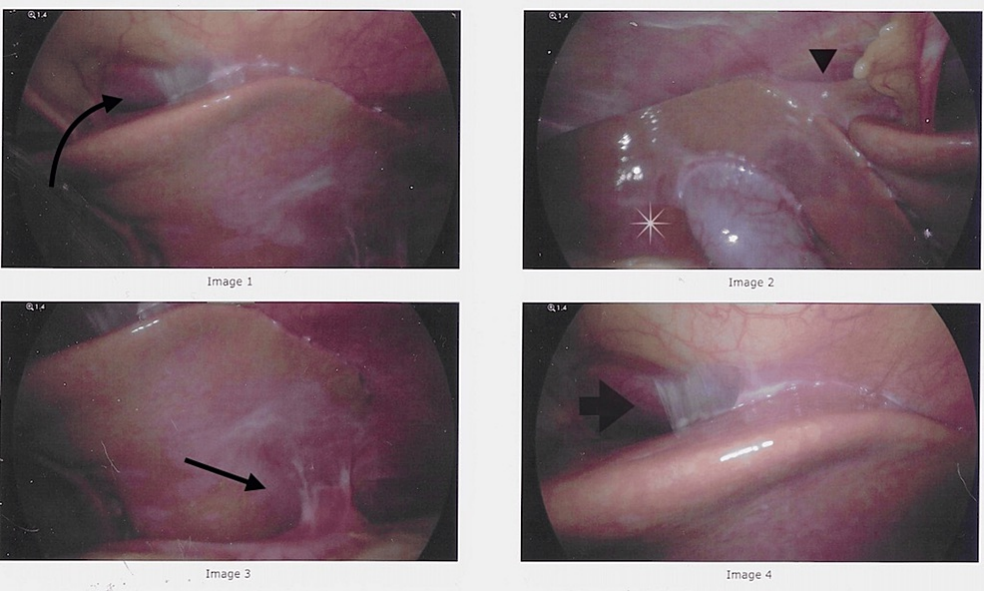
Intraoperative laparoscopic images of perihepatitis and contracted and intrahepatic gallbladder. Image 1: Perihepatic adhesions seen plastering the liver to the abdominal wall due to adhesions (arrow). Image 2: Retraction of the liver and intrahepatic gallbladder (asterisk) on the abdominal wall superiorly due to adhesions (arrowhead) despite patient positioning in reverse Trendelenburg. Image 3: Adhesive bands are seen on the inferior portion of the liver to the small bowel (arrow). Image 4: A thin veil-like adhesion seen on the superior liver border and abdominal wall (arrow).

**Figure 3:**
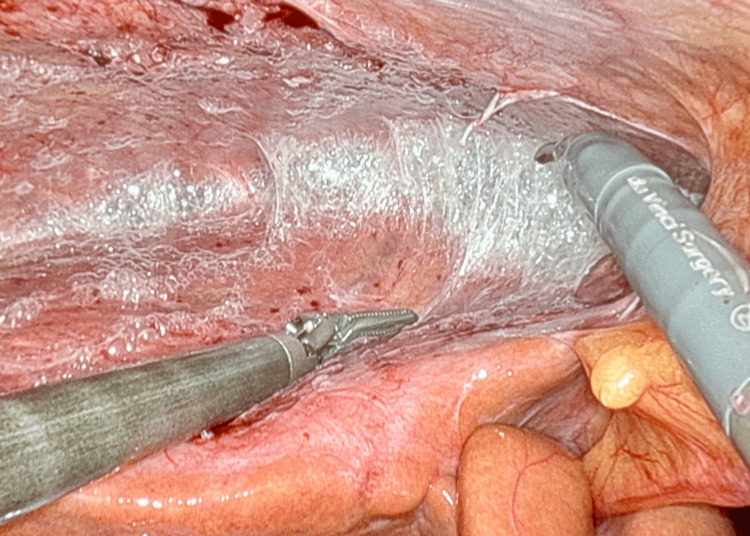
Intraoperative robotic images of perihepatitis. Perihepatic adhesions are taken down using robotic instruments seen under tension as violin string-like adhesions.

In the operating room, extensive perihepatic adhesions were seen across both lobes of the liver retracting the gallbladder superiorly (Figures 2, 3). Extensive lysis of adhesions was taken down with an additional 0.5 cm^2^ liver biopsy using a robotic monopolar curved scissor before the completion of difficult cholecystectomy given the extensive adhesions and contacted intrahepatic gallbladder. Pathology was significant for chronic cholecystitis with perihepatic mesothelial fibrosis with mild subcapsular hepatic steatosis with no evidence of liver fibrosis on trichrome and reticulin stains. On the two-week postoperative clinic visit, he reported a complete resolution of symptoms and was tolerating diet with no issues.

He was advised to follow up with his primary care provider for further evaluation and management of his sexual health.

## Discussion

FHCS was described by Thomas Fitz-Hugh and Arthur Curtis in the 1930s manuscript having three noteworthy findings: violin string-like adhesions spanning the anterior surface of the liver and anterior abdominal wall, a venereal disease, and a female patient. In that paper, three female patients; all in their 30s were thought to have some sort of biliary disease. Only one of the three patients had diagnostic laparotomy with drain placement, allowing peritoneal sampling to show gonorrhea, while the rest were diagnosed clinically [2,3].

It would then take 40 years before the first case of FHCS would be documented in a male patient. Lieutenant Kimball and Knee reported a 22-year-old man who presented with severe RUQ pain and a history of gonococcal urethritis. Liver biopsies showed *N. gonorrhoeae* with normal underlying liver tissue [4].

In 1978, Müller-Schoop demonstrated *C. trachomatis* in several patients who had undergone a diagnostic laparoscopy for peritonitis, six of whom also had perihepatitis. Historically, the main culprits have been *N. gonorrhoeae* and *C. trachomatis* with a ratio of 1:5. [5] Since then, several causes of FHCS have been reported from tuberculosis [6], an exaggerated immune response to chlamydia infection [7], and even after a peritoneal catheter removal [8]. In a retrospective single institutional study in South Korea, 100 obstetric cases of perihepatitis were found from an array of gynecological causes, from endometriosis, leiomyoma, gynecological malignancy, and PID [9].

It is theorized that the bacterial spread to the liver occurs either hematogenous or lymphatically in men versus ascending transperitoneal from the fallopian tubes via the right paracolic gutter in women. That may explain why bilobal hepatic involvement is more evident in male patients and right hepatic lobe predominance is seen in women as seen on contrast-enhanced CT scans on FHCS patients [10].

Disseminated gonococcal infection (DGI) is a rare complication of mucosal gonorrheal infection. DGI typically manifests two to three weeks after primary infection and affects approximately 0.5-3% of infected individuals causing cutaneous manifestation that affects the trunk, limbs, palms, and soles. Skin manifestations of DGI are variable; skin lesions are commonly described as non-pruritic, tender macules, papules, or pustules on a deeply erythematous or hemorrhagic base [11].

Retrospective studies of FHCS female patients’ emergency department visits have helped narrow the disease into two phases, namely, acute and chronic [12,13]. During the acute phase, patients present with episodic RUQ abdominal pain that may follow lower abdominal pain. They are associated with low-grade fevers and laboratory workup can show an elevated C-reactive protein and leucocytosis with characteristic pathologic findings of exudative hepatic capsule inflammation.

During the chronic phase, patients present with persistent dull RUQ abdominal pain that is refractory to several non-specific interventional therapies. They are less likely to present any constitutional symptoms, with antibody serology testing yielding more promising findings with specimen smears usually negative. On pathology, perihepatic exudative violin string-like adhesion is present between the hepatic capsule and abdominal wall.

Non-alcoholic fatty liver disease is present in 33% of the adult population in the United States [14]. It is defined as intrahepatic fat of at least 5% of liver weight. It is mainly a reactional response to stressful conditions, such as obesity, type 2 diabetes, and dyslipidemia. None of which the patient had per his laboratory workup. Its relationship to perihepatic fibrosis should be stressed not simply an “innocent bystander.”

The diagnosis of FHCS can be difficult because it may mimic many other diseases, including viral hepatitis, acute cholecystitis, pneumonia, pulmonary embolism, renal colic, and chronic pancreatitis. The diagnosis of FHCS requires a high level of suspicion, thorough history taking, including sexual and genitourinary history, and a detailed physical examination.

RUQ tenderness and spontaneous pain are elicited when the patient is placed in the left lateral recumbent position called the “liver capsule irritation sign.” Auscultation over the anterior costal margin may show a friction rub described as walking in new snow [15]. As blood and urine laboratory workup is mostly unremarkable, enhanced CT may demonstrate hepatic capsular enhancement that can aid in the diagnosis.

Mainline treatment is antibiotics if clinical suspicion is high, or lysis of perihepatic adhesions in cases resistant to antibiotics or diagnosed intraoperatively during a diagnostic laparoscopy.

## Conclusions

FHCS cases have classically been seen in women with an increasing trend of cases reported in men. Despite advancements in laboratory and radiological testing in the last century, FHCS remains elusive and is a diagnosis often made in the operating room. Further consideration should be given to the causes of FHCS other than the classic offenders, *N. gonorrhea* and *C. trachomatis*, and a better understanding of the phases of the disease can aid in earlier diagnosis and prevent its complications and unnecessary interventions.
